# Vanillin Production in *Pseudomonas*: Whole-Genome Sequencing of *Pseudomonas* sp. Strain 9.1 and Reannotation of Pseudomonas putida CalA as a Vanillin Reductase

**DOI:** 10.1128/AEM.02442-19

**Published:** 2020-03-02

**Authors:** Javier García-Hidalgo, Daniel P. Brink, Krithika Ravi, Catherine J. Paul, Gunnar Lidén, Marie F. Gorwa-Grauslund

**Affiliations:** a Applied Microbiology, Department of Chemistry, Lund University, Lund, Sweden; b Department of Chemical Engineering, Lund University, Lund, Sweden; c Water Resources Engineering, Department of Building and Environmental Technology, Lund University, Lund, Sweden; North Carolina State University

**Keywords:** NAD(P)H-dependent oxidoreductases, *Pseudomonas*, *calA*, *de novo* assembly, gene reannotation, vanillyl alcohol

## Abstract

Microbial degradation of lignin and its related aromatic compounds has great potential for the sustainable production of chemicals and bioremediation of contaminated soils. We previously isolated Pseudomonas sp. strain 9.1 from historical waste deposits (forming so-called fiber banks) released from pulp and paper mills along the Baltic Sea coast. The strain accumulated vanillyl alcohol during growth on vanillin, and while reported in other microbes, this phenotype is less common in wild-type pseudomonads. As the reduction of vanillin to vanillyl alcohol is an undesired trait in *Pseudomonas* strains engineered to accumulate vanillin, connecting the strain 9.1 phenotype with a genotype would increase the fundamental understanding and genetic engineering potential of microbial vanillin metabolism. The genome of *Pseudomonas* sp. 9.1 was sequenced and assembled. Annotation identified oxidoreductases with homology to Saccharomyces cerevisiae alcohol dehydrogenase *Sc*ADH6p, known to reduce vanillin to vanillyl alcohol, in both the 9.1 genome and the model strain Pseudomonas putida KT2440. Recombinant expression of the *Pseudomonas* sp. 9.1 FEZ21_09870 and P. putida KT2440 PP_2426 (*calA*) genes in Escherichia coli revealed that these open reading frames encode aldehyde reductases that convert vanillin to vanillyl alcohol, and that P. putida KT2440 PP_3839 encodes a coniferyl alcohol dehydrogenase that oxidizes coniferyl alcohol to coniferyl aldehyde (i.e., the function previously assigned to *calA*). The deletion of PP_2426 in P. putida GN442 engineered to accumulate vanillin resulted in a decrease in by-product (vanillyl alcohol) yield from 17% to ∼1%. Based on these results, we propose the reannotation of PP_2426 and FEZ21_09870 as *areA* and PP_3839 as *calA-II*.

**IMPORTANCE** Valorization of lignocellulose (nonedible plant matter) is of key interest for the sustainable production of chemicals from renewable resources. Lignin, one of the main constituents of lignocellulose, is a heterogeneous aromatic biopolymer that can be chemically depolymerized into a heterogeneous mixture of aromatic building blocks; those can be further converted by certain microbes into value-added aromatic chemicals, e.g., the flavoring agent vanillin. We previously isolated a *Pseudomonas* sp. strain with the (for the genus) unusual trait of vanillyl alcohol production during growth on vanillin. Whole-genome sequencing of the isolate led to the identification of a vanillin reductase candidate gene whose deletion in a recombinant vanillin-accumulating P. putida strain almost completely alleviated the undesired vanillyl alcohol by-product yield. These results represent an important step toward biotechnological production of vanillin from lignin using bacterial cell factories.

## Abstract (precis)

Valorization of lignocellulose (nonedible plant matter) is of key interest for the sustainable production of chemicals from renewable resources. Lignin, one of the main constituents of lignocellulose, is a heterogeneous aromatic biopolymer that can be chemically depolymerized into a heterogeneous mixture of aromatic building blocks; those can be further converted by certain microbes into value-added aromatic chemicals, e.g., the flavoring agent vanillin. We previously isolated a *Pseudomonas* sp. strain with the (for the genus) unusual trait of vanillyl alcohol production during growth on vanillin. Whole-genome sequencing of the isolate led to the identification of a vanillin reductase candidate gene whose deletion in a recombinant vanillin-accumulating P. putida strain almost completely alleviated the undesired vanillyl alcohol by-product yield. These results represent an important step toward biotechnological production of vanillin from lignin using bacterial cell factories.

## INTRODUCTION

Lignocellulosic biomass is a renewable feedstock of choice for the sustainable production of fine and bulk chemicals. Lignin, one of the three main components of lignocellulose together with hemicellulose and cellulose, is the most abundant aromatic biopolymer on Earth and thus a desirable feedstock for renewable aromatics (1, 2). Its high heterogeneity and recalcitrance have, however, limited its current industrial use, and large volumes of industrial lignin streams are incinerated for process energy rather than upgraded to value-added products (3, 4). From a biorefinery perspective, microbial bioconversion has emerged as a promising method for lignin valorization and gained much attention in recent years (5, 6). A common strategy is to combine the bioconversion with a pretreatment step where larger lignin polymers are broken down into smaller aromatic fragments by means of chemical depolymerization and then fed to microbes with relevant substrate specificities (7–9).

In nature, the conversion of depolymerized lignin is mainly performed by bacteria, although fungi and archaea have also been identified (10). While substrate specificities vary widely between species (and can even be strain dependent), the aromatic fragments are normally converted to monomers with a decreasing degree of substitution by means of so-called funneling pathways (11) ending with a ring fission that shunts the carbon into the central metabolism.

A number of bacteria capable of catabolism of lignin and/or lignin-derived aromatic compounds have been isolated (see, e.g., references 12–18). However, whereas characterization is a straightforward process, many isolates lack studies that elucidate the underlying molecular biology of their aromatic metabolic traits. We previously isolated Pseudomonas sp. strain 9.1 (DSM 105530) by culture-dependent screening of fibrous waste deposits from the pulp and paper industry collected on the northern Baltic coast in Sweden (19). The strain grew on seven out of 10 lignin model compounds tested as a sole carbon source (ferulate, *p*-coumarate, benzoate, vanillin, 4-hydroxybenzoate, vanillate, and vanillyl alcohol), whereas it could not sustain growth on syringate, guaiacol, or *cis*,*cis*-muconate (19). The present study continues the characterization of this isolate from a molecular point of view.

In comparison to a previous study on the *Pseudomonas* model organism P. putida KT2440 (20), *Pseudomonas* sp. 9.1 (here referred to as strain 9.1) displayed a distinct phenotype in that the strain excreted and reconsumed several intermediate metabolites of the lignin funneling pathways (19). The majority of the excreted substrates were metabolites produced directly downstream of the different substrates fed to the bacteria (e.g., 4-hydroxybenzoic acid from *p*-coumarate, and catechol and *cis*,*cis*-muconate from benzoic acid), which implies that certain reaction steps for aromatic assimilation in strain 9.1 are bottlenecks limiting the overall flux rate through the pathway. However, when grown on vanillin, the isolate excreted vanillyl alcohol, which is a less common trait for *Pseudomonas* spp. since they typically oxidize vanillin to vanillic acid as the first step for its assimilation. When grown on ferulate (a metabolite closely upstream of vanillin), no vanillyl alcohol was observed in strain 9.1 (19). The uptake rate of ferulate was very similar to that of vanillate, whereas vanillin was taken up much faster. Many microbes rapidly detoxify vanillin to its corresponding acid or alcohol to cope with its high reactivity (21). Given the above-described phenotype, it is likely that strain 9.1 relieves itself of the toxicity of vanillin by reducing it to vanillyl alcohol, which can then be later consumed once vanillin is depleted. While generally uncommon in the literature on pseudomonads, this particular phenotype has been reported in Pseudomonas fluorescens B56 (22) and certain fungi, e.g., Phanerochaete chrysosporium (23) and Saccharomyces cerevisiae (24).

Vanillin is one of the most utilized flavoring agents in food and fragrances and a precursor for the synthesis of polymers and fine chemicals (25). There is a high interest in replacing the chemical synthesis of vanillin from petro-based compounds (such as guaiacol) with biotechnological alternatives, such as production based on lignin (21, 25). A recent study on the engineering of P. putida KT2440 for bioconversion and accumulation of vanillin from ferulate reported issues with undesired vanillyl alcohol by-product formation (26). This is likely to also be the case for strain 9.1, meaning that elucidation of the enzymes responsible for the reduction of vanillin to vanillyl alcohol would not only contribute to an increased understanding of aromatic metabolism in *Pseudomonas* spp. but could also improve cell factory design for use in biorefineries (e.g., vanillin production). In the present study, the genome sequence of strain 9.1 was determined in order to relate the vanillyl alcohol-accumulating phenotype to a specific genotype. Experiments then based on this information were used to investigate the genetic basis of this phenotype, and together, these studies could be used to generate new *Pseudomonas* strains with alleviated vanillyl alcohol production.

## RESULTS

### Whole-genome sequencing and phylogeny.

The genome of *Pseudomonas* sp. 9.1 was sequenced with the aim of predicting the genetic basis of its aromatic funneling pathways and, in particular, to elucidate genetic clues regarding the oxidoreductase activities behind the vanillyl alcohol by-product formation. Whole-genome sequencing was performed with a paired-end Illumina MiSeq platform and a custom assembly pipeline designed using the SPAdes assembly algorithm (27) with iterative gap-filling steps (28–31). The final assembly consisted of 4,978,116 bp distributed over 30 contigs, with a GC content of 58.7%. This genome size was within the range for this genus, which is between 4 and 7 Mbp (Spiers et al. [32]). Statistics describing the sequencing, assembly and annotation are found in Table 1.

**TABLE 1:** Statistics of the sequencing, assembly, and annotation of *Pseudomonas* sp. 9.1 with Illumina MiSeq paired-end whole-genome sequencing^*a*^

Metric	Data
Sequencing and assembly results	
No. of paired-end reads	6,829,359
Mean read length	151 bp
Avg sequencing coverage	400×
No. of contigs	30
Total length (all contigs)	4,978,116 bp
Assembly *N*_50_	728,368 bp
Assembly *L*_50_	3
Assembly GC content	58.7%
Annotation results	
Total no. of genes	4,522
No. of CDSs with protein^*b*^	4,390
No. of RNA genes	75 (10 rRNAs, 61 tRNAs, 4 ncRNAs)^*c*^
No. of pseudogenes (without protein)	57

a The annotation was performed with the NCBI Prokaryotic Genome Annotation Pipeline.

b CDSs, coding sequences.

c ncRNAs, noncoding RNAs.

**FIG 1:**
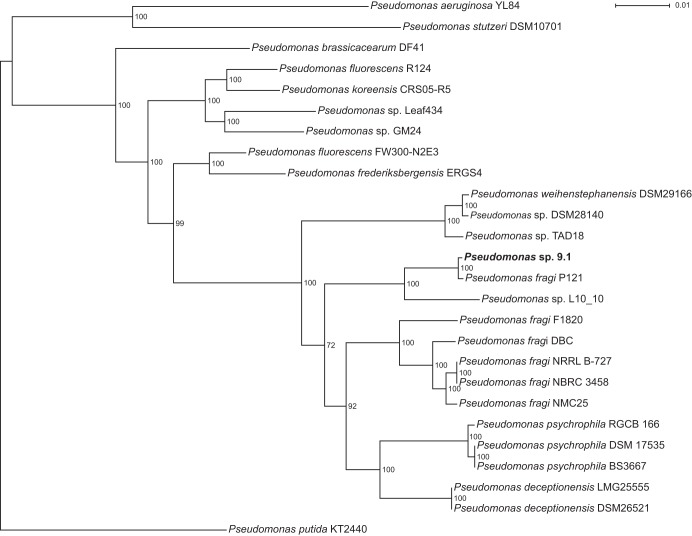
Phylogram of nonclinical *Pseudomonas* genomes in relation to strain 9.1, rooted at the model strain P. putida KT2440. Whole-genome phylogeny was calculated with the RealPhy pipeline, and trees were generated with RAxML (a maximum likelihood method). One hundred bootstrap iterations were used to construct the final tree. Bootstrap values are given at each branch. Strain 9.1 clearly clusters with other strains in the so-called *P. fragi* lineage (33). *Pseudomonas* sp. strain Lz4W clustered with *Pseudomonas* sp. 9.1 and *P. fragi* P121 throughout the analysis, but it had to be removed from the data set due to its high similarity to these strains resulting in bootstrap values below the 70% threshold (35).

Previously, strain 9.1 was tentatively identified by combined 16S rRNA and *gyrB* sequencing as Pseudomonas deceptionensis (19), which placed it in the P. fluorescens lineage and Pseudomonas fragi subgroup (33). To further resolve the taxonomy of strain 9.1, a genome-wide phylogeny approach (34), using multiple common loci across different *Pseudomonas* genomes and the 9.1 assembly, was used to calculate species relationships. Previous phylogeny studies have suggested that branches with >70% bootstrap support correspond to a 95% probability of representing a true clade (35), and this was therefore used as a threshold in the current project for selecting the final tree. Although it shared a common relative with *P. deceptionensis*, *Pseudomonas* sp. 9.1 rather clustered within the *P. fragi* taxon (Fig. 1) when more loci than just 16S rRNA and *gyrB* genes were considered. It was decided to no longer refer to the isolate as a *P. deceptionensis* as was previously suggested (19) but rather as *Pseudomonas* sp. 9.1.

The annotated 9.1 assembly was analyzed for open reading frames (ORFs) related to known aromatic funneling pathways commonly found in *Pseudomonas* spp. Known genes from the model strain P. putida KT2440 and a few other annotated species from the genus were used to predict the corresponding ORFs in strain 9.1 based on homology (see Table S1 in the supplemental material for BLASTp results). For almost every reaction, a clear gene candidate in 9.1 was predicted, with the exception of the catechol 1,2-dioxygenase *catA* gene, which had three highly similar hits. A number of strains of P. putida (including KT2440) have been reported to have two catechol 1,2-dioxygenases with functional redundancy (CatA and CatA2) in order to better cope with excess catechol levels (36, 37); it is likely that the multiple hits represent a similar arrangement of homologs in strain 9.1.

### Identification of putative oxidoreductases with activity on vanillin.

Saccharomyces cerevisiae ADH6p is an NADPH-dependent alcohol dehydrogenase with broad substrate range that, among other reactions, has been described to reduce vanillin to vanillyl alcohol (24). The amino acid sequence of *Sc*ADH6p was queried against the genome assemblies of strain 9.1 and the model strain P. putida KT2440 using stand-alone BLASTp. The best hit for strain 9.1 and the two best hits for KT2440 were selected as candidate genes; these were FEZ21_09870 in strain 9.1 and PP_2426 (*calA*) and PP_3839 (*adhP*) in KT2440 (Table 2). Analysis with the HMMER software (38) revealed that all three protein sequences for these genes contained the same functional domains as *Sc*ADH6 (Fig. S1), as follows: an N-terminal catalytic alcohol dehydrogenase GroES-like domain which should/could also act as the substrate-binding domain (ADH_N; Pfam accession no. PF08240) and a C-terminal zinc-containing domain presenting a classical Rossmann fold to bind NAD(P)H (ADH_zinc_N; Pfam accession no. PF00107). PP_2426 had been putatively annotated *in silico* as a gene for coniferyl dehydrogenase catalyzing the oxidation of coniferyl alcohol to coniferyl aldehyde (*calA*; the first reaction of the coniferyl branch), although this prediction was not experimentally validated (39).

**TABLE 2:** Top BLASTp results in strain 9.1 and the model strain Pseudomonas putida KT2440 with S. cerevisiae ADH6p as a query protein^*a*^

Strain	Gene	Identity (%)	Coverage (%)	E value	Locus annotation
*Pseudomonas* sp. 9.1	FEZ21_09870	40.5	92.2	1.0e−61	NAD(P)-dependent alcohol dehydrogenase
	FEZ21_15000	36.3	88.4	1.0e−59	NAD(P)-dependent alcohol dehydrogenase
P. putida KT2440	PP_2426 (*calA*)	39.3	87.3	2.9e−56	*calA* coniferyl alcohol dehydrogenase
	PP_3839 (*adhP*)	25.9	86.4	3.8e−27	*adhP* alcohol dehydrogenase

a Query protein number is SGDID:S000004937. Only the top hit was selected for strain 9.1 (FEZ21_09870).

### Oxidoreductase characterization in E. coli.

To assess the ability of the three selected candidate enzymes to reduce vanillin to vanillyl alcohol, the corresponding genes were amplified by PCR and introduced via plasmid pNIC28-Bsa4 to the expression host E. coli BL21(DE3). The resulting clones were grown in LB medium, and expression of the genes was induced by the addition of isopropyl-β-d-thiogalactopyranoside (IPTG). Overexpression of the recombinant genes as soluble proteins was confirmed by lysing cells by sonication and analyzing the resulting soluble intracellular extracts as well as the cell debris fractions by SDS-PAGE (Fig. S2).

**FIG 2:**
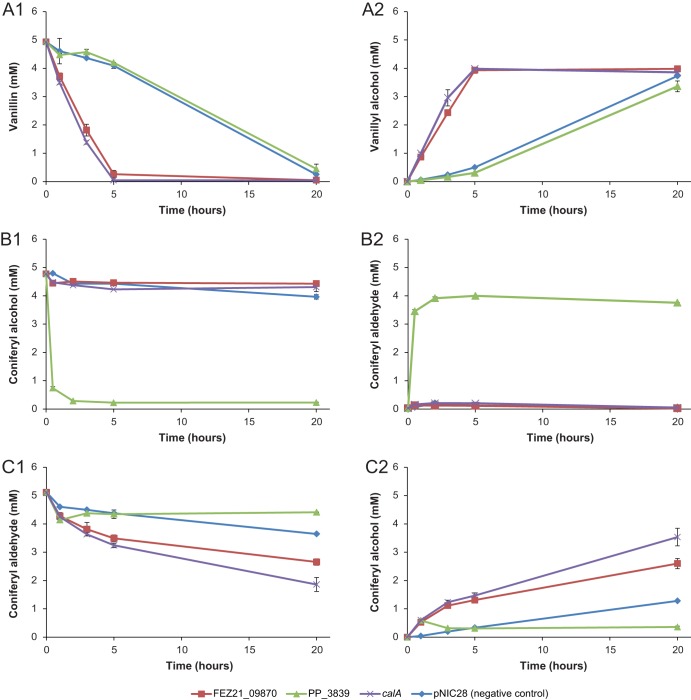
Whole-cell oxidoreductase assays with E. coli BL21(DE3) cells expressing the three candidate genes FEZ21_09870 (red squares), PP_3839 (green triangles), and *calA* (purple crosses) and the negative-control pNIC28-Bsa4KpnI (blue diamonds). (A to C) Three assays with different substrates were performed and evaluated with HPLC, as follows: A1 and A2 show concentrations of vanillin (substrate) and vanillyl alcohol (product), respectively; B1 and B2 show concentrations of coniferyl alcohol (substrate) and coniferyl aldehyde (product), respectively; and C1 and C2 show concentrations of coniferyl aldehyde (substrate) and coniferyl alcohol (product), respectively;. Experiments were performed in duplicates, and the standard deviations are displayed with an error bar.

Clones overexpressing each one of the candidate genes and the negative control [E. coli BL21(DE3) carrying the empty vector] were used in a whole-cell enzyme assay carried out in M9 medium supplemented with glucose to allow the regeneration of redox cofactors and 5 mM vanillin as the substrate (Fig. 2). The gene products of both *calA* and FEZ21_09870 presented very similar and clear vanillin reductase activities, with a complete conversion of vanillin into vanillyl alcohol in the first 5 h of the assay (Fig. 2A1 and A2). The reverse conversion was also assessed in separate assays with 5 mM vanillyl alcohol to evaluate the ability of these enzymes to oxidize vanillyl alcohol to vanillin. However, this activity was not detected from any of the strains tested (Fig. S3).

Since the functional analysis of P. putida KT2440 *calA* suggested that it could encode an enzyme able to oxidize coniferyl alcohol to coniferyl aldehyde (39), all three candidate enzymes were also evaluated for this reaction. Surprisingly, the gene products expressed from PP_2426 (*calA*) and FEZ21_09870 did not exhibit any activity on coniferyl alcohol (Fig. 2B1). The gene product from PP_3839, however, clearly displayed a very high oxidative activity on coniferyl alcohol, depleting the substrate in the first hour of the assay (Fig. 2B1 and B2). For the reverse reaction, enzymes encoded by PP_2426 (*calA*) and FEZ21_09870 instead exhibited some level of coniferyl aldehyde reductase activity (Fig. 2C1 and C2), i.e., the opposite reaction to the previously predicted function of PP_2426 (39). These results suggested that the genes PP_2426 and FEZ21_09870 encode aromatic aldehyde reductases *in vivo*, with broad substrate specificity.

While the FEZ21_09870 and PP_2426 gene products clearly reduced their substrates, there was additional background reductase activity on vanillin (Fig. 2A1 and 2) and coniferyl aldehyde (Fig. 2C1 and 2) in the E. coli BL21(DE3) clones expressing PP_3839 and in the negative control (empty plasmid). This is likely due to endogenous E. coli aldehyde reductases such as YahK, YjgB (*ahr*), and YqhD (40), which are present in the host strain BL21(DE3) genome (GenBank accession no. CP001509; Jeong et al. [41]). The lower rate of background activity compared to that observed in the clones expressing the FEZ21_09870 and PP_2426 gene products suggests that the enzymes responsible for the background activity are probably not specific for these substrates, and their level of expression is likely much lower than that of the overexpressed enzymes.

**FIG 3:**
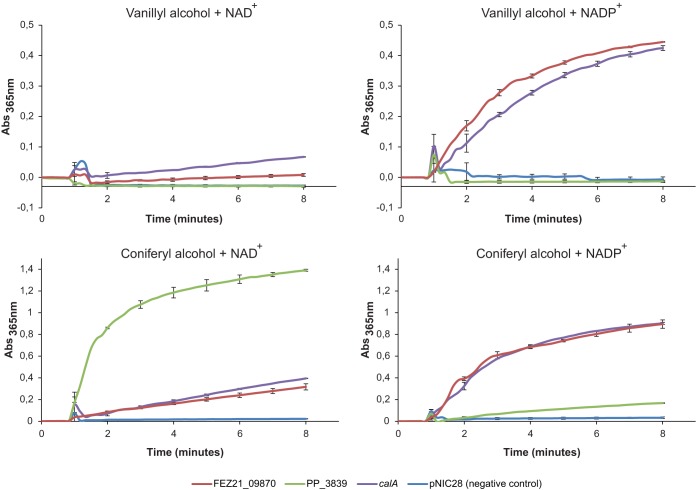
*In vitro* enzyme assays with intracellular extracts of E. coli BL21(DE3) cells expressing the three candidate genes FEZ21_09870 (red lines), PP_3839 (green lines), and *calA* (PP_2426) (purple lines) and the negative-control pNIC28-Bsa4KpnI (blue lines), with two different substrates (vanillyl alcohol and coniferyl alcohol) and two different redox cofactors (NAD^+^ and NADP^+^) in order to determine the preference of each enzyme for substrates and cofactors. Due to overlap in the absorbance (Abs) of products and reduced cofactors, the monitored absorbance was shifted from 340 to 365 nm, and no activity units were calculated. Experiments were performed in duplicate, and the standard deviations are displayed with error bars.

To determine the redox cofactor preference for each one of the candidate enzymes (encoded by FEZ21_09870, PP_2426 [*calA*], PP_3839), an *in vitro* spectrophotometric enzyme assay was developed with two different substrates, vanillyl alcohol and coniferyl alcohol. Both assayed reactions involve the oxidation of alcohols to aldehydes, given that oxidoreductases can catalyze their reactions in both directions depending on their architecture, substrate/product concentration, and cofactor availability. Activities were evaluated in the presence of NAD^+^ and NADP^+^. For these assays, soluble intracellular extracts of the E. coli BL21(DE3) clones overexpressing FEZ21_09870 and PP_2426 were employed. Both the PP_2426 and FEZ21_09870 gene products showed clear oxidation of vanillyl alcohol in the presence of NADP^+^ (Fig. 3), despite being the opposite reaction as observed in the *in vivo* whole-cell assays. They also showed low activity in the presence of NAD^+^ (Fig. 3). When assayed in the presence of coniferyl alcohol, the two enzymes confirmed their preference for NADP^+^, while presenting a certain degree of activity with NAD^+^ (Fig. 3). Taking together the outcomes of the whole-cell and *in vitro* assays, these results confirmed both enzymes as NADPH-dependent reductases.

The gene product of *adhP* (PP_3839) did not show any oxidative activity whatsoever with vanillyl alcohol as a substrate (Fig. 3), whereas coniferyl alcohol was rapidly oxidized in the presence of NAD^+^. A very low activity with NADP^+^ was also measured, being barely above that detected for the negative control.

### *adhP* gene (PP_3839) encodes a coniferyl alcohol dehydrogenase.

Enzyme assays performed with the gene product of PP_3839 (*adhP*) clearly pointed to a physiological role in coniferyl alcohol oxidation for this enzyme, which is precisely the function previously proposed for *calA* (PP_2426). The latter gene product, on the other hand, seemed to have a much higher reductase activity. To clarify this discrepancy, genomic deletion of both candidate genes was performed in the parental strain KT2440, followed by the analysis of the growth profile and substrate consumption of each deletion strain in the presence of 5 mM coniferyl alcohol.

**FIG 4:**
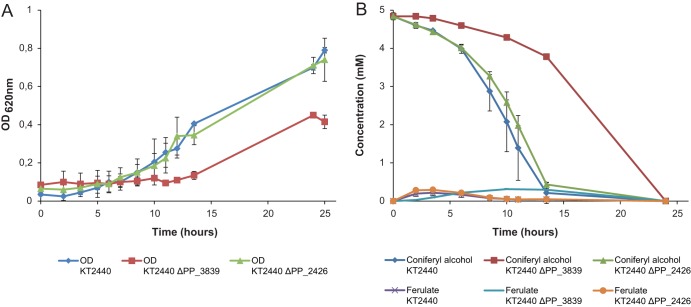
Fermentations of P. putida KT2440 and deletion strains with coniferyl alcohol 5 mM as the sole carbon source. (A) OD_620_ measurements of P. putida KT2440 (blue diamonds), P. putida KT2440 ΔPP_3839 (red squares), and P. putida KT2440 ΔPP_2426 (green triangles). (B) HPLC-determined concentration of coniferyl alcohol with P. putida KT2440 (dark-blue diamonds), P. putida KT2440 ΔPP_3839 (red squares), and P. putida KT2440 ΔPP_2426 (green triangles) and concentration of ferulate detected with P. putida KT2440 (purple crosses), P. putida KT2440 ΔPP_3839 (light-blue line), and P. putida KT2440 ΔPP_2426 (orange circles). Experiments were performed in duplicate, and the standard deviations are displayed with error bars.

The growth behavior of the strain carrying the *calA* deletion (KT2440 ΔPP_2426) under these conditions was almost identical to that of the wild-type strain (Fig. 4), consuming all of the substrate in the first 14 h, whereas the deletion strain KT2440 ΔPP_3839 showed a clearly different phenotype, with delayed growth and slower uptake of coniferyl alcohol. This result strongly supports the role of *adhP*, and not *calA*, as the gene encoding the main coniferyl alcohol dehydrogenase in P. putida KT2440, although it is clear that there are other alcohol dehydrogenases in this strain that are to oxidize this substrate with lower specificity.

### Deletion of PP_2426 in *Pseudomonas* decreases vanillyl alcohol formation.

Vanillin catabolism is operating at higher rates in P. putida KT2440 (4.87 mmol·gram of cell dry weight [g_CDW_]^−1^·h^−1^; Ravi et al. [20]) than in strain 9.1 (3.19 mmol·g_CDW_^−1^·h^−1^; Ravi et al. [19]), and vanillyl alcohol is not found as an excreted intermediate in KT2440. However, strain GN442, a KT2440-derived strain with deletions in genes directly and indirectly involved in the oxidation of vanillin, formed up to 15% (mol/mol) vanillyl alcohol during vanillin production from ferulate (26). This made GN442 an ideal background strain to evaluate the proposed vanillin reductase activity of the PP_2426 gene product.

**FIG 5:**
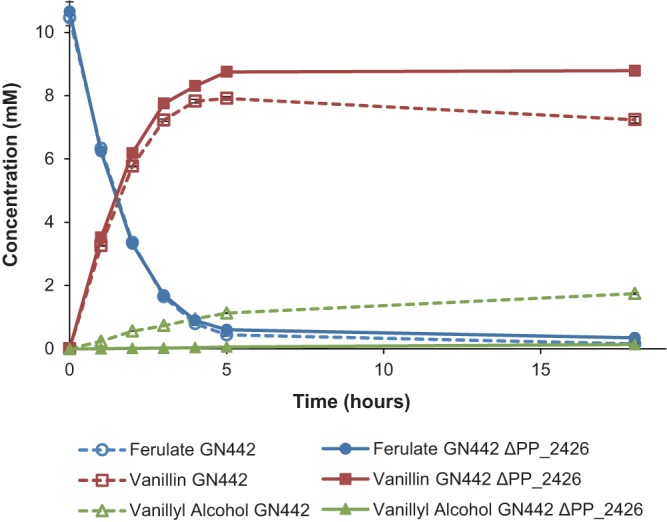
Bioconversion of ferulate into vanillin with the engineered P. putida strains GN442 (dashed lines) and GN442 *Δ*PP_2426 (solid lines). Concentrations of ferulate (blue circles), vanillin (red squares), and vanillyl alcohol (green triangles) were determined by HPLC. Experiments were performed in duplicate, and the standard deviations are displayed with error bars.

The formation of the vanillyl alcohol by-product was reproduced in P. putida GN442 using the established bioconversion protocol (26), with a final vanillyl alcohol yield of ∼17% (mol/mol) after 18 h, (dashed lines, Fig. 5). In the deletion strain (GN442 *Δ*PP_2426), however, the by-product formation was reduced to only ∼1% (mol/mol) vanillyl alcohol, detected after 18 h of bioconversion, and the vanillin yield increased from 69% in GN442 to 82% (mol/mol) in GN442 *Δ*PP_2426 (Fig. 5). The vanillin yield loss in GN442 appears to mainly result from the reduction of formed vanillin to vanillyl alcohol in the later stages of the process (Fig. 5).

## DISCUSSION

In the present study, whole-genome sequencing of *Pseudomonas* sp. 9.1, an isolate from lignin-rich waste material and that accumulates vanillyl alcohol from vanillin metabolism (19), led to the identification and reassessment of key genes participating in the metabolism of aromatic aldehydes. This knowledge was further used to obtain significant improvement in vanillin production from ferulate using genetic engineering.

The use of the fungal protein sequence from *Sc*ADH6p to query the 9.1 and KT2440 genomes for potential vanillin reductases that could explain the vanillyl alcohol formation proved to be a successful strategy. Contrary to the results from homology searches using genes/open reading frames associated with known participants in the funneling pathway (Table S1), the candidate open reading frames in the 9.1 and KT2440 genomes identified using the *Sc*ADH6p query only had sequence identities of around 25 to 40% (Table 2). It has been proposed as a rule of thumb that ≥30% sequence identity is a useful threshold for defining protein homology (42), although it has been debated that this cutoff might be too conservative and that E values are preferred (43). This study shows that low-identity hits should not be discarded when comparing proteins between different species, or belonging to different domains of life, as in the current case, between yeast and bacteria.

In contrast, the results from the experimental characterization of the three putative vanillin reductase genes, FEZ21_09870, *calA* (PP_2426), and *adhP* (PP_3839), highlighted that *in silico* predictions should be considered putative until experimental validation has been performed because they are, to a high degree, dependent on previous annotations in databases (44). Consequently, they are not as good at predicting less well-annotated reactions, such as the aromatic aldehyde reductions in the present case.

A main finding of the present study is that the function of the PP_2426 (*calA*) gene needs to be reannotated. One of the first descriptions of *calA* as a coniferyl alcohol dehydrogenase was from *Pseudomonas* sp. strain HR199 (45). However, the corresponding homologue in P. putida has been elusive; during the first genome sequencing project of KT2440, no *calA* orthologue was found (only *calB*, which catalyzes the next step in the pathway, being the conversion of coniferyl aldehyde to ferulate) (16, 46). It was only in a recent genome reassessment that a *calA* gene (PP_2426) was proposed in KT2440 (39); however, this prediction lacked experimental verification, and, as the present study has demonstrated, PP_2426 does not encode a coniferyl alcohol dehydrogenase but rather an aromatic aldehyde reductase able to reduce coniferyl aldehyde to coniferyl alcohol. Instead, it is P. putida KT2440 PP_3839 that should be annotated as a *cal* gene encoding coniferyl alcohol dehydrogenase activity, and we suggest the name *calA-II* in order not to cause conflict with older literature where PP_2426 was annotated as *calA*. We also suggest that the PP_2426 gene should be reannotated as *areA*, since the gene product from this open reading frame catalyzed the reduction of aromatic aldehydes to alcohols. It thus follows that FEZ21_09870 from *Pseudomonas* sp. 9.1 should also be annotated as Psp91*_areA*, as it displayed the same activity as PP_2426 (*areA*). The enzyme that allowed strain 9.1 to consume the accumulated vanillyl alcohol after vanillin was consumed (19) is yet to be identified. A summary of the findings and the proposed reannotations can be found in Table 3.

**TABLE 3:** Summary of the findings of the three candidate genes

Gene	Observations	Suggested reannotation
*Pseudomonas* sp. 9.1 FEZ21_09870	Clear reductase activity with vanillin and coniferyl aldehyde; no oxidizing activity with aromatic alcohols detected; confirmation of *in silico* predicted activity	Psp91*_areA* (vanillin reductase/aromatic aldehyde reductase gene)
P. putida KT2440 PP_2426 (*calA*)	High sequence identity to FEZ21_09870 (82.3%; BLASTp); identical activity to FEZ21_09870; unexpected reductase activity on coniferyl aldehyde, instead of the proposed oxidative activity (39); reannotation needed	*areA* (vanillin reductase/aromatic aldehyde reductase gene)
P. putida KT2440 PP_3839 (*adhP*)	No activity with aromatic aldehydes; rapid conversion of coniferyl alcohol into coniferyl aldehyde; likely the true P. putida coniferyl alcohol dehydrogenase, a function previously incorrectly attributed to PP_2426	*calA-II* (coniferyl alcohol dehydrogenase gene)

**FIG 6:**
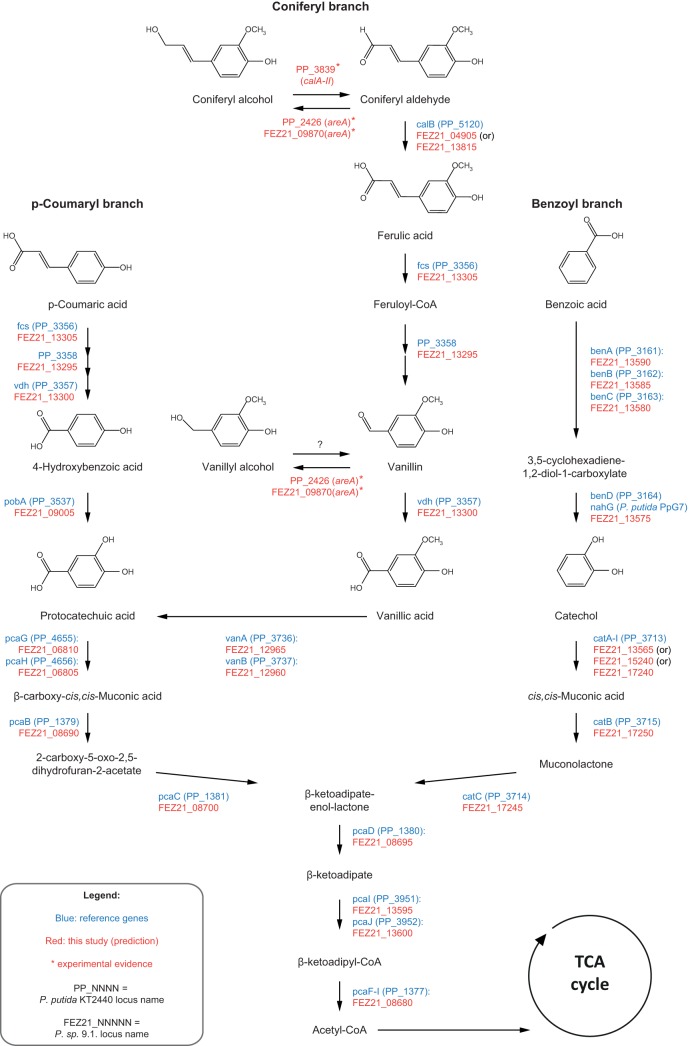
*In silico* predictions for the aromatic funneling pathway genes in *Pseudomonas* sp. 9.1 and P. putida KT2440. Growth has previously been demonstrated for strain 9.1 on ferulic acid, *p*-coumaric acid, benzoic acid and vanillin (19), which suggests that known P. putida funneling pathways are also likely to be found in 9.1. The suggested reannotations of the three candidate genes evaluated in this study (FEZ21_09870, PP_2426, and PP_3839) are referred to by the suggested reannotations (cf. the Discussion). The reaction in strain 9.1 to consume the accumulated vanillyl alcohol is unknown, and thus, the proposed reaction to oxidize it back to vanillin is denoted by a question mark. CoA, coenzyme A; TCA, tricarboxylic acid.

Using the new information about the functionality of the three candidate proteins, we propose an improved map of the aromatic funneling pathways in P. putida KT2440 (Fig. 6; gene names beginning with PP). The figure (Fig. 6) also contains the result of the analysis of the annotation of strain 9.1 (gene names beginning with FEZ21). The systematic locus names of the genes show that many of the funneling pathway genes in 9.1 are found in close proximity within the genome (just like in other *Pseudomonas* species [16]), suggesting that they belong to putative operons (Fig. S4). The annotation of FEZ21_09870 (Psp91*_areA*) as an aldehyde reductase made us wonder whether there was a PP_3839 (*calA-II*) gene equivalent in the strain 9.1 genome. Although strain 9.1 has not been assayed for growth on coniferyl alcohol, the growth on ferulate (downstream in the funneling branch) and the putative hits for *calB* in the assembly (Fig. 6) hinted to the possibility of a coniferyl alcohol dehydrogenase phenotype. The top BLASTp hits for PP_3839 (*calA-II*) equivalent in the 9.1 genome were FEZ21_15000 and FEZ21_09870 (Psp91*_areA*) (Tables 2 and S1), the latter having already been demonstrated to catalyze the reverse reaction. FEZ21_15000 was the second highest hit for *Sc*ADH6p in 9.1 (Table 2), suggesting the possibility that it encodes an oxidoreductase, although as we have demonstrated in the current study, experimental assessment is required to propose a more specific physiological role for FEZ21_15000.

While the NAD(P)H-binding motif GxGxxG/A (47, 48) is present in the three investigated proteins and the reference protein (*Sc*ADH6), any further extrapolation on cofactor usage was not possible from sequence analysis alone because accurate distinction of NADH sites from NADPH sites from a given primary polypeptide sequence is nontrivial (49). It is widely assumed that NAD(H)-dependent proteins are generally related to oxidative metabolic processes (48, 50), and NADP(H)-dependent proteins are involved in the reduction of xenobiotic carbonyls in yeasts (51, 52) and in lignin biosynthesis in plants (53, 54). In the current study, the *calA-II* (PP_3839) gene product involved in the assimilation of coniferyl alcohol was indeed NAD^+^ dependent, whereas PP_2426 (*areA*) and FEZ21_09870 (Psp91_*areA*) were mostly NADPH dependent and indeed fulfilled a detoxification role.

One of the few other studies on *Pseudomonas* spp. that mentions the same vanillyl alcohol phenotype as strain 9.1 concerns the engineering of P. putida KT2440 to accumulate vanillin from ferulate; the authors were unable to find a deletion that could alleviate vanillyl alcohol by-product formation (26). We applied our findings about PP_2426 (*areA*) to their final strain GN442 and were able to demonstrate that the GN442 *Δ*PP_2426 strain presented greatly decreased vanillyl alcohol formation compared to that of the parental strain (17% and 1%, respectively). This also shows that there is little to no background aldehyde reductase activity in KT2440 that acts on vanillin once PP_2426 (*areA*) has been deleted, which reinforces the proposed functional reannotation of this gene, and by extension Psp91*_areA* (FEZ21_09870), which shares the same activity.

Besides helping us determine the *in vivo* role of PP_2426, the resulting mutant strain GN442 *Δ*PP_2426 represents an interesting platform for the conversion of ferulate into vanillin with very high yield and short conversion times. This specific result is also a valuable advance for any endeavor aiming for the biological production of vanillin or related aldehydes using engineered *Pseudomonas* strains or any other microbial platform.

The present study demonstrates that the characterization of novel isolates not only provides information about the diversity of metabolic phenotypes in nature but also describes how this information can be applied to address unresolved traits of model strains. By investigating the peculiar trait of *Pseudomonas* sp. 9.1 to accumulate vanillyl alcohol during growth on vanillin (only to consume it again after vanillin is depleted), we have identified the gene encoding the aldehyde reductase in this strain (FEZ21_09870). PP_2426 (here proposed to be reannotated as *areA*) in P. putida KT2440 had the same activity on vanillin, a trait that is completely masked in wild-type KT2440 due to different tuning of activities in the coniferyl funneling pathway branch compared to strain 9.1. This finding resulted in the improved KT2440-derived cell factory (GN442 *Δ*PP_2426) designed to overproduce vanillin by the deletion of PP_2426 (*areA*). Additionally, we proved that PP_3839, unspecifically annotated as alcohol dehydrogenase gene *adhP*, is the gene encoding the dehydrogenase responsible for the oxidation of coniferyl alcohol to coniferyl aldehyde (a function inaccurately attributed to the gene product of PP_2426). We propose the reannotation of this gene as coniferyl alcohol dehydrogenase gene *calA-II*.

This study thus reinforces the importance of exploring novel isolates together with comparisons to the well-understood model organisms. A strain that may be undesirable as an industrial cell factory (e.g., in terms of rates and yields compared to the model strain) may reveal, through targeted genome sequence analysis, novel traits that can both contribute valuable knowledge to the understanding of the genus and lead to improved engineered strains for diverse biotechnological applications.

## MATERIALS AND METHODS

### Genome extraction and sequencing.

*Pseudomonas* sp. isolate 9.1 (DSM 105530; Ravi et al. [19]) and P. putida KT2440 (DSM 6125) were cultivated in liquid LB medium (Sambrook and Russell [55]; see “Culture media and conditions,” below), and genomic DNA from strain 9.1 was extracted using the Invitrogen PureLink genomic DNA minikit (Thermo Scientific, Carlsbad, CA, USA) and eluted with Invitrogen UltraPure DNase/RNase-free distilled water (Thermo Fisher Scientific, Waltham, MA, USA). Genomic DNA from P. putida KT2440 was purified using the GeneJET genomic DNA purification kit from Thermo Fisher Scientific Baltics (Vilnius, Lithuania). Paired-end whole-genome sequencing was performed using an Illumina MiSeq (2 × 150 bp) platform at GATC Biotech AG (Cologne, Germany). The raw data from the sequencer consisted of 6,829,359 paired reads with an average read length of 151 bp and 58% GC content (Table 1). Read quality was assessed with FastQC (v0.11.5; Andrews [56]).

### *De novo* assembly.

The raw reads were subjected to quality trimming with sickle (v1.210; Joshi and Fass [57]), followed by an additional round of quality control with FastQC. *De novo* assembly of the trimmed reads was performed with SPAdes (v3.11.1; Bankevich et al. [27]), and assembly metrics were analyzed with QUAST (v4.5.4; Gurevich et al. [58]). To improve the assembly, a custom iterative gap-filling pipeline was designed where the assembly and trimmed reads were in turn subjected to SSPACE_Standard (v3.0; Boetzer et al. [31]) with bwa (v0.7.17; Li and Durbin [59]), AlignGraph (28) with bowtie2 (v2.3.3.1; Langmead and Salzberg [60]) and blat (v34; Kent [61]), and GapFiller (v1.10; Boetzer and Pirovano [29]) and ABySS-sealer (v2.0.2; Paulino et al. [30]). QUAST was used to measure assembly improvements after each iteration of the pipeline; after three iterations of gap filling, the improvement in assembly metrics reached stagnation, and thus, it was decided to proceed with that version of the assembly. Contigs were sorted using Mauve (v2015-02-13; Darling et al. [62]) in a biologically relevant order using the closely related Pseudomonas deceptionensis LMG 25555 assembly (GenBank accession number GCA_900106095.1) as a reference genome. The final assembly was annotated using the NCBI Prokaryotic Genome Annotation Pipeline (PGAP) (63).

### Phylogeny.

Genome-wide phylogeny was performed with a set of genomes from 108 nonclinical isolates in the *Pseudomonas* genome database (Winsor et al. [64]). The number of genomes was, however, reduced in an iterative process to find trees with good statistical basis (i.e., removing genomes that were too similar to their closest relatives). The phylogeny was calculated using RealPhy (v1.12; Bertels et al. [34]), which was run with bowtie2 (v2.3.3.1; Langmead and Salzberg [60]) and SAMtools (v1.6; Li et al. [65]) as a read mapper. Strain 9.1 was compared to the other genomes using fastq paired-end reads after trimming with sickle (see “*De novo* assembly,” above). The set of genomes that was used in the final version of the RealPhy analysis used 26 genomes (including strain 9.1) and inferred phylogeny from 4,223 patterns out of a total of 53,115 polymorphic sites in the set. All trees were built with the maximum likelihood method using RAxML (v8.2.10; Stamatakis [66]) with the GTRGAMMA model and 100 bootstrap iterations. Dendroscope (v3.5.9; Huson and Scornavacca [67]) was used to visualize the phylogenetic trees.

### Identification of funneling pathways and putative oxidoreductases.

Stand-alone BLASTp using the NCBI Genome Workbench (v2.12.10) was used to compare known funneling pathway proteins from *Pseudomonas* species to the genome of strain 9.1. The *Pseudomonas* sp. 9.1 assembly was converted into a stand-alone BLAST database with makeblastdb (BLAST v2.7.1). Query protein amino acid sequences (Table S1) were downloaded from NCBI GenBank (68). The amino acid sequence of S. cerevisiae S288c ADH6p was taken from The *Saccharomyces* Genome Database (https://www.yeastgenome.org/) (69). Putative vanillin reductases with homology to *Sc*ADH6p were identified with the same stand-alone BLASTp procedure using the 9.1 assembly as well as the latest version of the P. putida KT2440 genome (GenBank accession no. AE015451.2) (39). The HMMER Web server (https://www.ebi.ac.uk/Tools/hmmer/) was used to analyze the candidate proteins for structural features (38), and protein domain data were taken from the Pfam database (http://pfam.xfam.org/) (El-Gebali et al. [70]).

### Culture media and conditions.

E. coli BL21(DE3) (71) cells were routinely cultured in lysogeny broth (LB) medium (10 g/liter tryptone, 5 g/liter yeast extract, 10 g/liter NaCl) at 37°C. *Pseudomonas* strains were cultured in LB medium or in mineral M9 medium (55) supplemented with trace element solution (72) and appropriate carbon sources at 30°C. Antibiotics were added to the media when necessary at the following concentrations: 150 μg/ml ampicillin, 50 μg/ml kanamycin, and 30 μg/ml chloramphenicol; the concentration of ampicillin was increased to 500 μg/ml for *Pseudomonas* strains. IPTG was added at 1 mM to induce the expression of genes under the T7 promoter (pNIC28 plasmid) or 5 mM to induce the expression of the *ech-fcs* operon controlled by the *tac* promoter in P. putida GN442 and GN442 ΔPP_2426 strains before the bioconversion assays. All the strains and plasmids used in the study are listed in Table 4.

**TABLE 4:** List of plasmids and strains used in the current study

Strain or plasmid	Features or genotype^*a*^	Reference
Bacterial strains		
E. coli DH5α λ*pir*	Strain used for preparation and conjugative delivery of pSEVA212S-derived plasmids; F^−^ ϕ*dlacZ*ΔM15 Δ*lacZYA-argF U169 deoR supE44 hsdR17 recA1 endA1 gyrA96 thi-1 relA1*	Dunn et al. (78)
E. coli HB101	Helper strain carrying pRK600 plasmid, used in triparental conjugation; F^−^ *thi-1 hsdS20* (r_B_^–^, m_B_^–^) *supE44 recA13 ara-14 leuB6 proA2 lacY1 galK2 rpsL20* (Str^r^) *xyl-5 mtl-1*	Boyer and Roulland-Dussoix (79)
E. coli BL21(DE3)	Strain used for high-level T7 expression of cloned oxidoreductases; *fhuA2* [*lon*] *ompT gal* (*λDE3*) [*dcm*] *ΔhsdS λ sBamHIo Δ*EcoRI-B *int*::(*lacI*::*PlacUV5*::*T7 gene1*) *i21 Δnin5*	Studier and Moffatt (71)
Pseudomonas putida KT2440	Model organism	DSM 6125
P. putida KT2440 ΔPP_2426	Deletion mutant without *calA* (PP_2426) ORF	This study
P. putida KT2440 ΔPP_3839	Deletion mutant without PP_3839 ORF	This study
P. putida GN442	Engineered strain for production of vanillin from ferulate; Δ*upp* ΔPP_0166-0168 Δ*vdh* ΔPP_3827-3832 ΔPP_2680 ΔPP_0545 ΔPP_1948 *lacI*^q^-*P_tac_-ech-fcs*	Graf and Altenbuchner (26)
P. putida GN442 ΔPP_2426	Optimized strain for high-yield production of vanillin from ferulate; Δ*upp* ΔPP_0166-0168 Δ*vdh* ΔPP_3827-3832 ΔPP_2680 ΔPP_0545 ΔPP_1948 *lacI*^q^-*P_tac_-ech-fcs* ΔPP_2426	This study
*Pseudomonas* sp. 9.1	Environmental isolate	Ravi et al. (19), DSM 105530
Plasmids		
pNIC28-Bsa4	Plasmid for ligation-independent cloning and T7 expression of cloned genes, Km^r^	Savitsky et al. (80)
pNIC28-Bsa4KpnI	KpnI-digested and religated version of pNIC28-Bsa4 used as negative-control plasmid, Km^r^	This study
pNIC28-Bsa4-*calA*	Clone of PP_2426 in pNIC28-Bsa4, Km^r^	This study
pNIC28-Bsa4-PP_3839	Clone of PP_3839 in pNIC28-Bsa4, Km^r^	This study
pNIC28-Bsa4-FEZ21_09870	Clone of FEZ21_09870 in pNIC28-Bsa4, Km^r^	This study
pSEVA212S	Suicide plasmid for integration in *Pseudomonas* genomes, Km^r^	Martínez-García and de Lorenzo (75)
pSEVA212S-Δ*calA*	pSEVA212S containing the upstream and downstream regions of *calA* assembled together, Km^r^	This study
pSEVA212S-ΔPP_3839	pSEVA212S containing the upstream and downstream regions of PP_3839 assembled together, Km^r^	This study
pRK600	Helper plasmid used in triparental mating between *E, coli* and *Pseudomonas* strains, Cm^r^	Finan et al. (81)
pSEVA128S	Plasmid carrying the gene encoding I-SceI endonuclease, Ap^r^	Martínez-García and de Lorenzo (75)

a Str^r^, streptomycin resistance; Km^r^, kanamycin resistance; Cm^r^, chloramphenicol resistance; Ap^r^, apramycin resistance.

### Shake flask fermentations.

Seed cultures of P. putida KT2440 (DSM 6125), P. putida KT2440 ΔPP_3839, and P. putida KT2440 ΔPP_2426 were grown overnight in 10 ml M9 medium with 10 g/liter glucose in 50-ml conical tubes. Cells from these seed cultures were harvested, washed with 0.9% sterile saline solution, and used to inoculate 250-ml shake flasks with 20 ml M9 medium with 5 mM coniferyl alcohol at an optical density at 620 nm (OD_620_) of around 0.1. Flasks were further incubated at 30°C with 200 rpm orbital shaking. Samples were taken at different time points for OD_620_ measurement and high-performance liquid chromatography (HPLC) analysis. All fermentations were carried out in duplicate.

### Ferulate bioconversion assays.

Bioconversion assays of ferulate to vanillin were performed with strains P. putida GN442 (26) and P. putida GN442 ΔPP_2426, as described by Graf and Altenbuchner (26), with slight modifications. Briefly, overnight LB cultures of both strains were diluted 50 times and further cultured in LB shake flasks to generate biomass, with overexpression of the enzymes responsible for the conversion of ferulate (Fcs and Ech) induced by the addition of IPTG. After this phase, the OD_620_ was measured, and cells were harvested, aiming for a final OD_620_ of 7.2 (approximately 25 × 10^9^ cells). These cells were gently washed with 50 mM sterile sodium phosphate buffer (pH 7.2) and resuspended in 5 ml of the same buffer containing 10 mM ferulate in 50-ml conical tubes. Tubes were shaken at 200 rpm and 30°C for 18 h; samples were taken at several time points for HPLC analysis.

### Cloning and expression of recombinant oxidoreductases.

Candidate genes *calA* (PP_2426) and PP_3839 from P. putida KT2440 and FEZ21_09870 from *Pseudomonas* sp. 9.1 were amplified by PCR with Phusion high-fidelity DNA polymerase (Thermo Fisher Scientific), according to the instructions of the manufacturer, using the primers calA LIC Fw and calA LIC Rv, PP_3839 LIC Fw and PP_3839 LIC Rv, and peg1955 LIC Fw and peg1955 LIC Rv, respectively (see full primer list and sequences in Table S2). The resulting DNA fragments were purified using the GeneJET PCR purification kit (Thermo Fisher Scientific) and sent for sequencing with the same primers to Eurofins Genomics (Ebersberg, Germany). Verified fragments were inserted into BsaI-linearized pNIC28-Bsa4 plasmid by ligation-independent cloning with the In-Fusion HD cloning kit (Clontech, Mountain View, CA, USA) and introduced into chemically competent E. coli BL21(DE3) cells. In order to generate a negative-control plasmid not producing levansucrase SacB, pNIC28-Bsa4 was digested with KpnI and then religated to lose the negative selection marker *sacB*, obtaining the plasmid pNIC28-Bsa4KpnI, which was also introduced into E. coli BL21(DE3).

For the overexpression of the cloned genes, overnight LB cultures of the corresponding E. coli BL21(DE3) clones and negative-control strain E. coli BL21(DE3)/pNIC28-Bsa4KpnI were diluted to an OD_620_ of 0.1 in 30 ml fresh LB medium with kanamycin and further incubated at 37°C until reaching OD_620_ of around 0.6, and then 1 mM IPTG was added and incubation was extended for 5 additional hours. Cells were subsequently harvested and washed with sterile 0.9% NaCl and resuspended in 1 ml lysis buffer (25 mM Tris-HCl [pH 8] with NaCl 100 mM) prior to their lysis by sonication with a Branson Sonifier 150 equipped with a microprobe. Finally, lysed cells were centrifuged at 4°C and 21,130 × *g* for 20 min, and supernatants were collected as soluble intracellular extracts for SDS-PAGE analysis and *in vitro* enzyme assays. SDS-PAGE analysis of cell extracts was performed in a 4 to 20% Mini-Protean TGX precast gel (Bio-Rad, Hercules, CA, USA) using a PageRuler prestained 10- to 180-kDa protein ladder (Thermo Fisher Scientific Baltics, Vilnius, Lithuania) as a molecular mass marker.

### Whole-cell oxidoreductase assays.

E. coli BL21(DE3) cells overexpressing the cloned genes from *Pseudomonas* spp. as well as the negative-control strain E. coli BL21(DE3)/pNIC28-Bsa4KpnI were prepared from 5 ml of LB culture as described in “Culture media and conditions” above. Following the wash with saline solution, cells were resuspended in 5 ml of M9 medium with 50 μg/ml kanamycin, 1 mM IPTG, 10 g/liter d-glucose, and 5 mM aromatic substrates (vanillin, vanillyl alcohol, coniferyl aldehyde, or coniferyl alcohol). These cells were incubated at 30°C and 250 rpm orbital shaking for 20 h; samples were taken at several time points for HPLC analysis. All assays were done in duplicate.

### *In vitro* enzyme assays.

Relative alcohol dehydrogenase activity of the different overexpressed enzymes in E. coli BL21(DE3) soluble intracellular extracts was assessed in the presence of two different substrates (vanillyl alcohol and coniferyl alcohol) with two different redox cofactors (NAD^+^ and NADP^+^) by a spectrophotometric assay based on that described by Larroy et al. (73). Assays were carried out in duplicate in a total volume of 600 μl at 30°C in an Ultrospec 2100 pro UV-Vis spectrophotometer (GE Healthcare, Little Chalfont, UK) equipped with a Peltier cell holder. The reaction mixture contained 33 mM sodium phosphate buffer (pH 7) with 1 mM substrate and 0.5 mM cofactor. Reactions were started by adding the corresponding cell extract (40 μg total protein) after 1 min of baseline reading. Progression of the reactions was followed by monitoring the absorbance at 365 nm every 10 s for 8 min.

### Genomic deletions in *Pseudomonas* strains.

Genomic deletions of individual genes in P. putida KT2440 and P. putida GN442 were carried out following previous protocols (74–76). Briefly, the adjacent regions of the target genes were amplified by PCR, assembled by overlap-extension PCR (see list of primers used in Table S2), and cloned into the suicide plasmid pSEVA212S carrying a kanamycin resistance gene and two sites for the homing endonuclease I-SceI. The resulting plasmids were introduced and maintained in E. coli DH5α λ*pir* and subsequently transferred to a recipient *Pseudomonas* strain by triparental mating using the aforementioned E. coli strain together with the helper strain E. coli HB101/pRK600. Since this plasmid cannot replicate in *Pseudomonas* spp., the kanamycin resistance can only be acquired by chromosomal integration of the whole plasmid. After this event, the plasmid pSEVA128S containing ampicillin resistance and the gene encoding the endonuclease I-SceI were delivered by electroporation to a kanamycin-resistant *Pseudomonas* culture, leading to the generation of two lethal double-strand breaks which must be repaired by RecA-dependent homologous recombination with the adjacent regions inserted in the pSEVA212S plasmid. Electroporation of P. putida electrocompetent cells was done according to the protocols of Martínez-García and de Lorenzo (74), using 0.2-cm-wide gap cuvettes with a Gene Pulser apparatus equipped with a pulse controller (Bio-Rad, Hercules, CA, USA). Deletion of the genes had to be confirmed by PCR amplification and sequencing of the surrounding region with the primers calA Fw, calA Rv, PP_3839 Fw, and PP_3839 Rv (Table S2). Finally, the plasmid pSEVA128S was cured by several consecutive passes on LB without antibiotic until the ampicillin resistance was lost. Verified mutant strains were stored at −80°C until further use.

### Chromatographic analysis.

The culture aliquots were centrifuged (20,000 × *g*) for 3 min, and the supernatants were filtered before analysis. A Waters Acquity HPLC system coupled with a UV detector (Milford, MA, USA) was used for the analysis of aromatic compounds. An Agilent InfinityLab Poroshell 120 EC-C_18_ column with an internal diameter of 4.6 mm, length of 100 mm, and 4-μm particle size was used for separation. The column heater was set to 50°C. The mobile phases consisted of water and acetonitrile, with acetic acid as an additive. A gradient elution method previously described (77) was used for analysis. The sample injection volume was 5 μl. Peaks were quantified using the area under the curve against their authentic calibration standards.

### Data availability.

The sequence reads from this article have been deposited at the NCBI Sequence Read Archive under the accession no. PRJNA543218. The assembly data set supporting the results of this article has been deposited at DDBJ/EMBL/GenBank under the accession no. VBWF00000000. The version described in this paper is VBWF00000000.

## Supplementary Material

Supplemental file 1
