# Supplementary material for: Vanillin Production in Pseudomonas: Whole-Genome Sequencing of Pseudomonas sp. Strain 9.1 and Reannotation of Pseudomonas putida CalA as a Vanillin Reductase
Source: Appl Environ Microbiol. 2020 Mar 2;86(6):e02442-19. doi: 10.1128/AEM.02442-19 (PMC7054097; doi:10.1128/AEM.02442-19)
Supplement: Supplemental file 1 [file AEM.02442-19-s0001.pdf]

Supplemental Information for

Bacterial isolate genome annotation as a driver for improved microbial cell factories: *calA* from *Pseudomonas putida* encodes a vanillin reductase

Javier García-Hidalgo[1], Daniel P Brink[1], Krithika Ravi[2], Catherine J. Paul[1,3], Gunnar Lidén[2] and Marie F Gorwa-Grauslund[1]*

[1]Applied Microbiology, Department of Chemistry, Lund University, P.O. Box 124, SE-221 00 Lund, Sweden

[2]Department of Chemical Engineering, Lund University, P.O Box 124, 221 00 Lund, Sweden

[3]Water Resources Engineering, Department of Building and Environmental Technology, Lund University, P.O Box 118, SE-221 00 Lund, Sweden

*Corresponding author

E- mail: Marie-Francoise.Gorwa@tmb.lth.se

Supplemental Figures

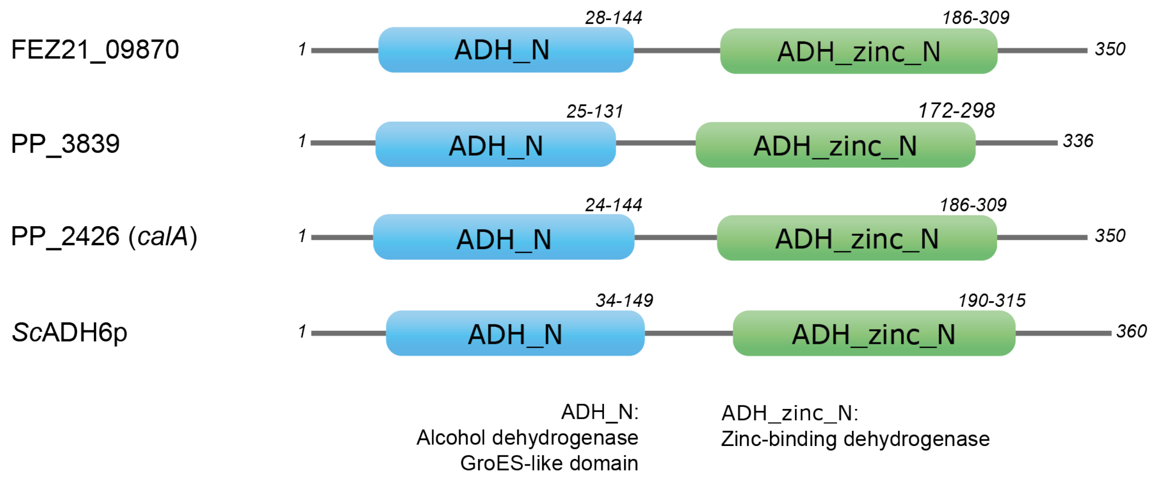

Figure S1. Sequence features/domains in the three candidate proteins and the reference protein *Sc*ADH6p predicted by HMMER. The numbers represent the number of amino acids (aa) from the start of the polypeptide. Total protein length (aa) is presented after each polypeptide. Descriptions of the ADH_N and ADH_zinc_N domains can be found at the Pfam database at accession numbers PF08240.12 and PF00107.26, respectively.

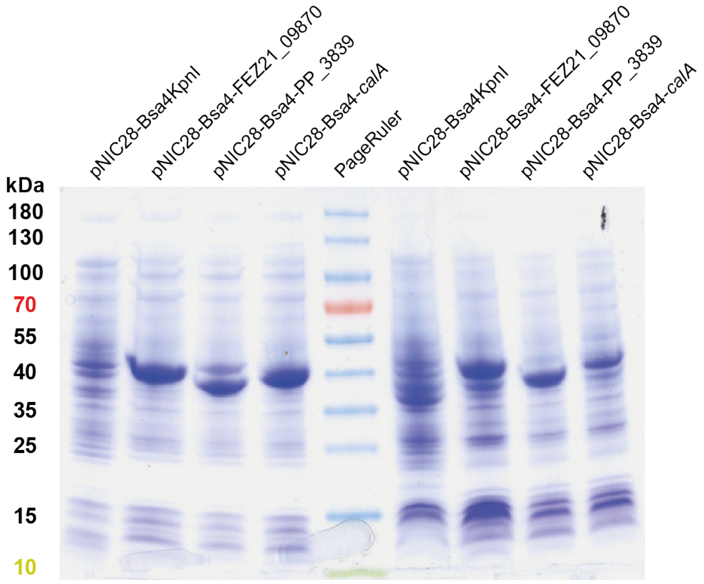

Figure S2. SDS-PAGE analysis of *E. coli* BL21(DE3) cells overexpressing the cloned genes FEZ21_09870, PP_3839 and *calA* (PP_2426), plus the empty plasmid control pNIC28-Bsa4KpnI. Intracellular soluble extracts are shown on the left side of the PageRuler and insoluble cell debris are shown on the right side.

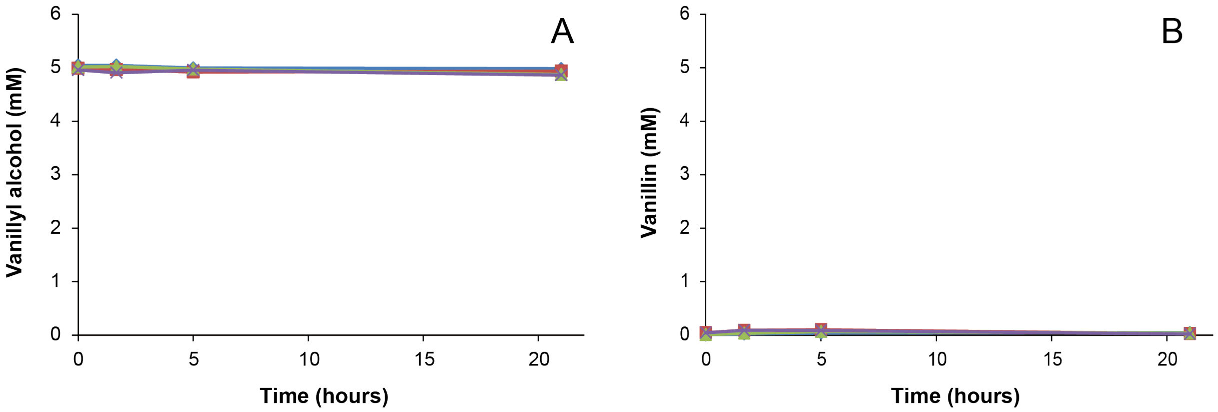

Figure S3. Whole-cell oxidoreductase assay with *E. coli* BL21(DE3) cells expressing the three candidate genes FEZ21_09870 (red squares), PP_3839 (green triangles) and *calA* (PP_2426) (purple crosses) and the negative control pNIC28-Bsa4KpnI (blue diamonds). The assay with vanillyl alcohol is depicted: **A** shows concentrations of vanillyl alcohol (substrate) and **B** shows concentration of vanillin (product). Experiments were performed in duplicates and the standard deviations are displayed with an error bar.

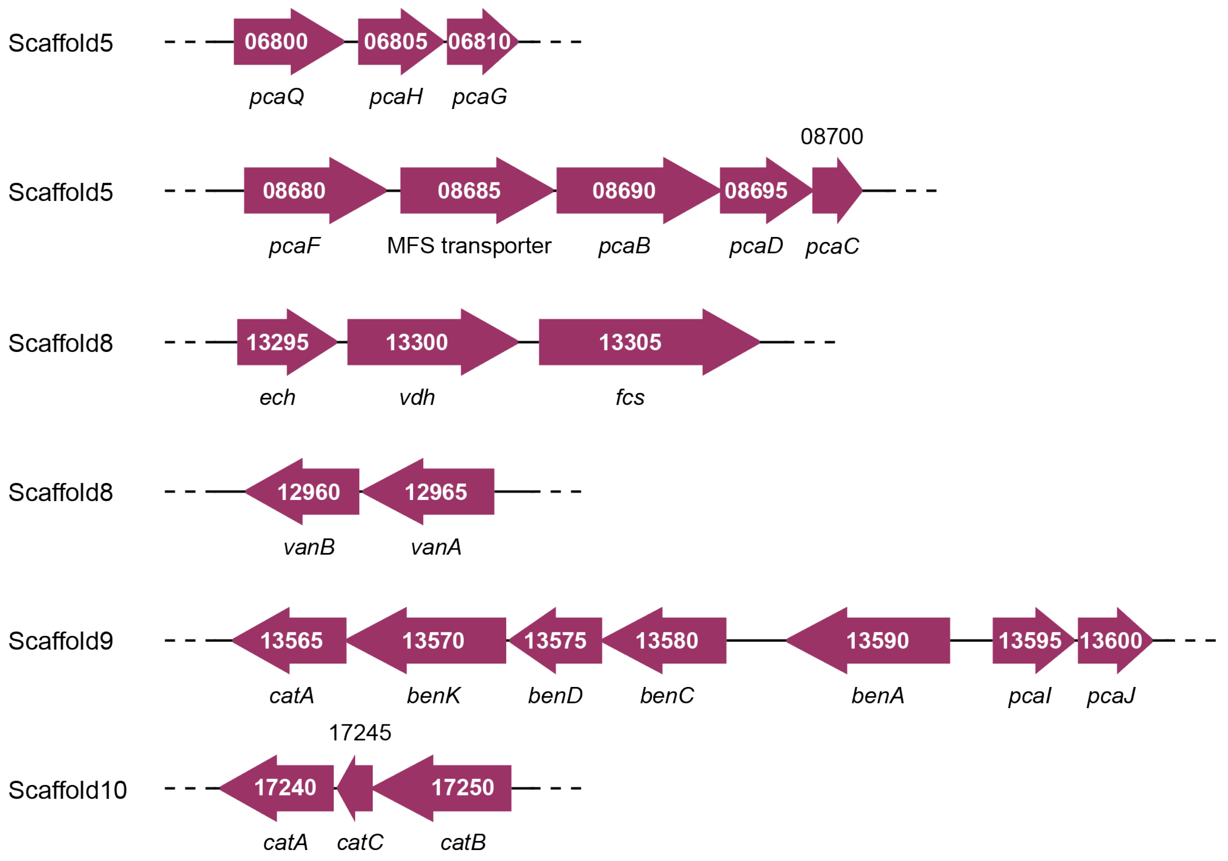

Figure S4. Gene clusters in *Pseudomonas* sp. 9.1 related to aromatic funneling pathways (see Figure 6). The loci IDs have been stripped of the *FEZ21_* prefix for simplicity. The gene annotations are based on homology to *P. putida* KT2440 genes and should as such be considered putative pending experimental verification. The different scaffolds are related to Figure 6 by containing putative genes for the following reactions: Scaffold5: conversion of protocatechuate to β-ketoadipate; Scaffold8: conversion of ferulic acid to protocatechuic acid; Scaffold9: conversion of benzoic acid to catechol, and β-ketoadipate to acetyl-CoA; Scaffold10: conversion of catechol to β-ketoenoladipate.

Supplemental Tables

Table S1. Query proteins used to assess the aromatic funneling pathways and related reactions in strain 9.1 with BLASTp. The results of this table were used to construct the pathway map in Figure 6. Proteins with systematic names beginning with PP_ come from the model strain *P. putida* KT2440. Contrary to *P. alcaligenes* NCIB 9867 and *P. putida* KT2440, isolate 9.1 did not have any homologous gene products related to the gentisate branch.

Query protein [GenBank Accession No.]	Resulting protein in strain 9.1	Identity (%)	Coverage (%)	e-value	Locus annotation (strain 9.1)
PP_3356 (*fcs*) [NP_745496.2]	FEZ21_13305	77.0	99.8	0	feruloyl-CoA synthase
P. putida T2 *ech* [CAD60261.1]	FEZ21_13295	86.9	100	4.0e-159	*p*-hydroxycinnamoyl-CoA hydratase/lyase
PP_3358 (*ech* homolog) [AAN68962.1]	FEZ21_13295	92.8	100	0	*p*-hydroxycinnamoyl-CoA hydratase/lyase
PP_3357 (*vdh*) [NP_745497.1]	FEZ21_13300	87.3	100	0	aldehyde dehydrogenase
PP_3336 (*vanA*) [NP_745868.1]	FEZ21_12965	87.7	98.9	0	aromatic ring-hydroxylating dioxygenase subunit alpha
PP_3337 (*vanB*) [NP_745869]	FEZ21_12960	77.2	100	0	oxidoreductase
PP_4655 (*pcaG*) [NP_746764.1]	FEZ21_06810	81.1	100	1.6e-122	pcaG: protocatechuate 3,4-dioxygenase subunit alpha

PP_4656 (*pcaH*) [NP_746765.1]	FEZ21_06805	86.6	100	3.8e-161	pcaH: protocatechuate 3,4-dioxygenase subunit beta
PP_2426 (*areA,* formerly *calA*) [NP_744574.1]	FEZ21_09870	82.3	100	0	NAD(P)-dependent alcohol dehydrogenase
PP_5120 (*calB*) [NP_747221.1]	FEZ21_04905	68.2	96.6	0	coniferyl aldehyde dehydrogenase
	FEZ21_13815	45.5	94.1	9.2e-131	coniferyl aldehyde dehydrogenase
PP_1379 (*pcaB*) [NP_743538.1]	FEZ21_08690	78.3	99.6	0	3-carboxy-*cis,cis*-muconate cycloisomerase
PP_1381 (*pcaC*) [NP_743540.1]	FEZ21_08700	89.2	100	2.6e-86	pcaC: 4-carboxymuconolactone decarboxylase
PP_1380 (*pcaD*) [NP_743539.1]	FEZ21_08695	69.2	97.7	2.3e-132	pcaD: 3-oxoadipate enol-lactonase
PP_3951 (*pcaI*) [NP_746081.1]	FEZ21_13595	86.9	96.1	1.9e-136	3-oxoacid CoA-transferase subunit A
PP_3952 (*pcaJ*) [NP_746082.1]	FEZ21_13600	88.3	100	8.0e-142	CoA transferase subunit B
PP_1377 (*pcaF-I*) [NP_743536.1]	FEZ21_08680	88.5	100	0	pcaF: 3-oxoadipyl-CoA thiolase
PP_3280 (*paaJ)* [AAN68887.1]	FEZ21_08680	67.9	98.0	0	pcaF: 3-oxoadipyl-CoA thiolase
	FEZ21_15940	53.3	97.8	5.3e-138	3-oxoadipyl-CoA thiolase
PP_3713 (*catA-I*) [NP_745846.1]	FEZ21_13565	73.1	99.4	1.3e-173	catA: catechol 1,2-dioxygenase
	FEZ21_15240	67.5	99.0	3.7e-159	catA: catechol 1,2-dioxygenase
	FEZ21_17240	65.1	99.4	3.3e-146	catA: catechol 1,2-dioxygenase

PP_3715 (*catB*) [NP_745848.1]	FEZ21_17250	76.9	99.7	0	muconate cycloisomerase
PP_3714 (*catC*) [NP_745847.1]	FEZ21_17245	76.6	100	1.5e-51	catC: muconolactone Delta-isomerase
PP_3161 (*benA*) [NP_745305.1]	FEZ21_13590	85.2	100	0	benA: benzoate 1,2-dioxygenase large subunit
PP_3162 (*benB*) [NP_745306.1]	FEZ21_13585	87.7	100	2.5e-106	benB: benzoate 1,2-dioxygenase small subunit
PP_3163 (*benC*) [NP_745307.1]	FEZ21_13580	79.2	100	0	2Fe-2S iron-sulfur cluster binding domain-containing protein
PP_3164 (*benD*) [NP_745308.1]	FEZ21_13575	83.0	99.6	3.3e-153	1,6-dihydroxycyclohexa-2,4-diene-1-carboxylate dehydrogenase
PP_3165 (*benK*) [NP_745309.1]	FEZ21_13570	78.4	100	0	MFS transporter
PP_3537 (*pobA*) [NP_745674.1]	FEZ21_09005	73.4	99.5	0	pobA: 4-hydroxybenzoate 3-monooxygenase
PP_2216 (*acd*) [AAN67829.1]	FEZ21_13680	84.0	100	0	acyl-CoA dehydrogenase
PP_4005 (*aat*) [AAN69599.1]	FEZ21_11180	76.9	99.6	1.3e-131	Leucyl/phenylalanyl-tRNA--protein transferase
P. putida PpG7 *nahG* AAA25897.1	FEZ21_15235	70.6	92.6	0	salA: salicylate 1-monooxygenase

PP_0074 (*aroE*)<br>[NP_742244.1]	FEZ21_03700	77.1	95.6	1.2e-153	aroE: shikimate dehydrogenase
PP_4621 (*hmgA*)<br>[AAN70194.1]	FEZ21_21850	82.3	100		homogentisate 1,2-dioxygenase
PP_4620 (*hmgB*)<br>[AAN70193.1]	FEZ21_21845	78.8	99.8	0	fahA: fumarylacetoacetase
PP_4619 (*hmgC*)<br>[AAN70192.1]	FEZ21_21840	72.2	99.5	1.4e-110	maiA: maleylacetoacetate isomerase
PP_3569 (*quiA*)<br>[AAN69170.1]	FEZ21_15275	77.1	97.8	0	glucose/quinate/shikimate family membrane-bound PQQ-dependent dehydrogenase
PP_0560 (*aroQ-I*)<br>[AAN66187.1]	FEZ21_01030	93.3	98.7	9.0e-100	aroQ: type II 3-dehydroquinate dehydratase
PP_2407 (*aroQ-II*)<br>[AAN68019.1]	FEZ21_01030	59.3	96.6	2.3e-53	aroQ: type II 3-dehydroquinate dehydratase
PP_3003 (*aroQ-III*)<br>[AAN68611.1]	FEZ21_01030	47.2	99.3	5.8e-45	aroQ: type II 3-dehydroquinate dehydratase
PP_2554 (*quiC*)<br>[NP_744699.1]	FEZ21_06825	80.7	99.7	0	TIM barrel protein
PP_3839 (*calA-II)*	FEZ21_15000	29.3	92.3	0	NAD(P)H-dependent alcohol dehydrogenase
	FEZ21_09870<br>(Psp91_*areA*)	29.9	95.5	0	NAD(P)H-dependent alcohol dehydrogenase
PP_2518 (*gllIA*)<br>[AAN68130.2]	No hit				

PP_2515 (*gllB*) [AAN68127.1]	No hit				
PP_2514 *gllC*) [AAN68126.1]	No hit				
PP_2513 (gllD) [AAN68125.1]	FEZ21_09480	36.2	96.4	6.9e-55	prpF: 2-methylaconitate cis-trans isomerase PrpF
P. alcaligenes NCIB 9867 *xlnE* [AAD49427.1]	No hit				
P. alcaligenes NCIB 9867 *hbzE* [ABD64514.1]	No hit				
P. alcaligenes NCIB 9867 *MhpD* [ABD64512.1]	No hit				

Table S2. List and features of PCR primers used in this work. Oligonucleotide tails for ligation-independent cloning are shown in italics, overhangs for overlapping extension PCR are shown in bold, restriction sites introduced for restriction cloning (EcoRI and BamHI) are underlined.

Name	Length	Sequence (5´→3´)	Application
calA LIC Fw	34	*TACTTCCAATCCATG*TATACCGCCATCGGTTATG	Cloning of PP_2426
calA LIC Rv	33	*TATCCACCTTTACTG*TCAGCCCTGTAGCGTGGC	
PP_3839 LIC Fw	34	*TACTTCCAATCCATG*AAAGCTGCTGTCGTTGCAC	Cloning of PP_3839
PP_3839 LIC Rv	36	*TATCCACCTTTACTG*TCAGCCTTCGAACTGTATCAC	
peg1955 LIC Fw	34	*TACTTCCAATCCATG*TACAACGCCATCGGTTATG	Cloning of FEZ21_09870
peg1955 LIC Rv	39	*TATCCACCTTTACTG*TTAAGCCTTCAGGGTTTTCATGTC	
calA a	30	CGGAATTCGCTCGCGCAGGTGGGCGAGGGC	Deletion of PP_2426
calA b	45	**CCCGTACAGGCAACCGATCAA**GCAGGGCCTCGCAGTGAAGTGAAA	
calA c	21	TTGATCGGTTGCCTGTACGGG	
calA d	28	CGGGATCCCTTCGTACTGGCCACTCAAG	
PP_3839 a	30	CGGAATTCCGCGCTGTTCGTGCTGGGCACG	Deletion of PP_3839
PP_3839 b	45	**CACATGACCTGGCTCGGCCTG**GAGAGCCTCCGTGTCTATCCGATG	
PP_3839 c	21	CAGGCCGAGCCAGGTCATGTG	
PP_3839 d	29	CGGGATCCGAAAGGTGACACCGCAGATAA	
calA Fw	20	CGATGCCCACCGCCACGCCC	Verification of PP_2426 deletion
calA Rv	23	GTGCCACGCTGGCCGTGAGGGTG	
PP_3839 Fw	22	GAGAAATTCGTGGTCAAGCAGG	Verification of PP_3839 deletion
PP_3839 Rv	21	GTCTTCAGCATGAAGGATGAG	
